# Perception and safety analysis of COVID‐19 vaccination in cancer patients: A multicenter, real‐world study

**DOI:** 10.1002/cam4.5400

**Published:** 2022-11-13

**Authors:** Kyoungmin Lee, In Hae Park, Sang Cheul Oh, Jae Hong Seo, Min Ji Jeon, Eun Sang Yu, Dae Sik Kim, Chul Won Choi, Ah‐reum Lim, Myung Han Hyun, Ju Won Kim, Jwa Hoon Kim, Yoon Ji Choi, Soohyeon Lee, Kyong Hwa Park, Yeul Hong Kim, Jung Yoon Choi, Jung Sun Kim, Se Ryeon Lee, Hwa Jung Sung, Eun Joo Kang

**Affiliations:** ^1^ Division of Hemato‐Oncology, Department of Internal Medicine, Korea University College of Medicine Korea University Guro Hospital Seoul Republic of Korea; ^2^ Division of Hemato‐Oncology, Department of Internal Medicine, Korea University College of Medicine Korea University Anam Hospital Seoul Republic of Korea; ^3^ Division of Hemato‐Oncology, Department of Internal Medicine, Korea University College of Medicine Korea University Ansan Hospital Ansan Republic of Korea

**Keywords:** cancer patients, coronavirus disease 2019, perception, safety, vaccine

## Abstract

**Background:**

Although various coronavirus disease 2019 (COVID‐19) vaccines have been delivered to the public worldwide, data on cancer populations are limited. Vaccine hesitancy related to safety concerns is observed among cancer patients. We report the perception of COVID‐19 vaccines and their safety profile after vaccination among cancer patients.

**Materials and Methods:**

Between April and November 2021, a multicenter survey was conducted on 318 patients treated in any hemato‐oncology outpatient clinic among three hospitals under the Korea University Medical Center. The medical records of the patients were reviewed to obtain detailed clinical and hematological toxicity data.

**Results:**

A perception survey was conducted among 293 patients. Among them, 53.9% were concerned about developing vaccine‐related adverse events (VRAEs) and 23.5%, about negative effects on cancer treatment. During the study period, 255 and 186 patients participated in a safety survey after the first and second doses, respectively. After the first dose, 62% of patients reported VRAEs (2.4%, grade 3), whereas 48.9% reported VRAEs (2.7%, grade 3) after the second dose. For both doses, injection‐site pain and sore arm pain were the most common VRAEs, followed by myalgia, fatigue, and headache. No grade 4/5 VRAEs were observed, and there were no differences in complete blood count after vaccination. Multivariate analysis revealed female sex, active cancer treatment, and mRNA vaccines as independent risk factors for VRAE development in cancer patients.

**Conclusion:**

Despite high levels of concern, COVID‐19 vaccines were well tolerated by cancer patients, with a safety profile consistent with that of the general population.

## Graphical abstract

Our study indicated that COVID‐19 vaccines were well tolerated and no severe adverse effects were observed in cancer patients, while there was high prevalence of fear related to vaccine‐associated adverse effects. Based on our results, we should reassure patients about the safety of COVID‐19 vaccines.

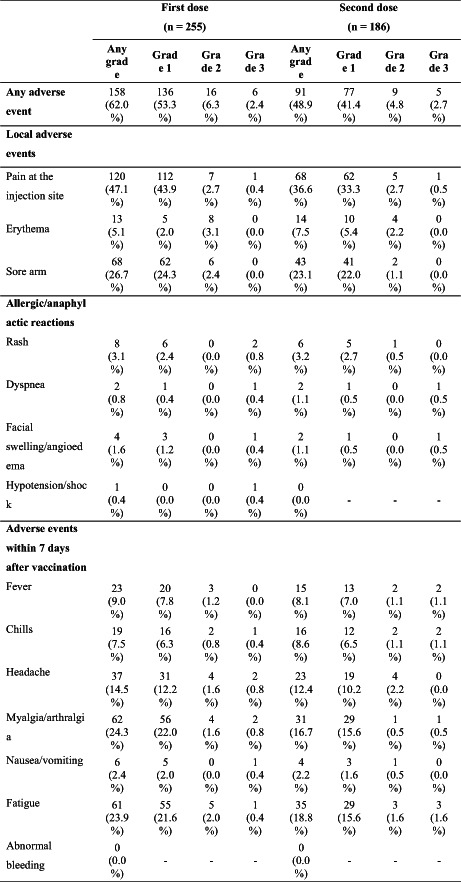

## 1 INTRODUCTION

Since the first outbreak of coronavirus disease 2019 (COVID‐19) in December 2019, the novel coronavirus (SARS‐CoV‐2) has infected more than 300 million people and claimed 5 million lives worldwide to date.^1^ In this pandemic, the damage to high‐risk groups, such as the elderly and those with underlying diseases, is inevitably greater; a pooled analysis showed increased mortality from COVID‐19 in patients with malignancies.^2^ Indeed, compared to the general population, patients with cancer presented atypical symptoms or asymptomatic infections that mask the actual COVID‐19 infection,^3^, ^4^ but had a higher rate of mechanical ventilation and COVID‐19‐related mortality.^5^ While there is no definitive cure for COVID‐19, effective vaccines have been rapidly developed.^6^ Various COVID‐19 vaccines have been delivered to the public, and, given their vulnerability, cancer patients are considered a priority for vaccination.^7^

However, owing to the ineligibility of cancer patients in most trials at the time of vaccine development, there is a scarcity of data on the adverse effects of COVID‐19 vaccines in patients with active malignancies.^8^ This has raised safety concerns among patients, leading to an increase in fear and hesitancy toward vaccines. In fact, a study conducted on more than 1000 cancer patients in South Korea revealed that only 61.2% of the patients were willing to be vaccinated.^9^ Fortunately, with the widespread application of approved COVID‐19 vaccines in some countries, significant volumes of data on efficacy and safety of the vaccines for patients with cancer are now emerging, alleviating these concerns.^10^, ^11^, ^12^, ^13^

COVID‐19 vaccination in South Korea has been ongoing since February 2021, and all residents in South Korea aged 12 years or more are currently eligible for vaccination. The Oxford‐AstraZeneca vaccine was the first vaccine to be approved and introduced, followed by the Pfizer/BioNTech, Janssen, and Moderna vaccines. As of December 30, 2021, 44.16 million people have received more than one dose, and the vaccination rate relative to the population was 86.0% for the first, 82.7% for the second, and 33.4% for the third doses in South Korea.^14^

In this study, we surveyed the perception of COVID‐19 vaccination among patients with cancer in Korea and collected data on vaccine‐related adverse events (VRAEs) to evaluate the real‐world safety of COVID‐19 vaccines in patients with hemato‐oncologic diseases.

## 2 MATERIALS AND METHODS

In Korea, vaccination was started in the high‐risk group on February 26, 2021. The average daily number of confirmed cases was 470 in the 1st quarter of 2021. However, this gradually increased to 590 in the 2nd, 1680 in the 3rd, and 3470 in the 4th quarter as new variants emerged. In Korea, the Delta variant was dominant from July 2021 to December 2021, and the Omicron variant became dominant from January 2022. Overall, the total number of confirmed cases of COVID‐19 in Korea is approximately 570,000, with approximately 1100 confirmed cases per 100,000 population in 2021.^15^

### 2.1 Study Population and Questionnaire

A multicenter survey was conducted between April and November 2021 on 318 patients treated in any hemato‐oncology outpatient clinic at three hospitals under the Korea University Medical Center. To obtain real‐world data from a broad range of patients with cancer, all patients who consented to participate in the survey were included in this study. The study participants were first asked to complete a questionnaire regarding their perceptions of COVID‐19 vaccination. Participants who had already been vaccinated on the day of consent were asked to complete a post‐vaccination questionnaire, and those who had not yet been vaccinated at that time completed the questionnaire on outpatient visits after their first and/or second vaccine dose. The postvaccination survey was conducted separately for the first and second vaccine doses. The questionnaire was in Korean. The perception questionnaire was prepared after review by all oncologists and hematologists of the three hospitals, and the post‐vaccination questionnaire was prepared based on items reported in published clinical trials of vaccines.

The perception questionnaire comprised two parts with seven questions: willingness to be vaccinated against COVID‐19 and attitude toward COVID‐19 and COVID‐19 vaccines in relation to cancer treatment. Patients were asked if they were willing to receive the COVID‐19 vaccination (“yes” or “no” or “not yet decided”), the factors that most influenced their decision to get vaccinated (“my own thought” or “national recommendation” or “physicians' recommendation” or “family or friends' recommendation”) and what concerned them the most (“types of vaccine” or “efficacy” or “side effects” or “effects on cancer treatment”). In addition, patients were asked about the risk and prognosis of COVID‐19 infection in cancer patients compared with the general population, the risk of COVID‐19 infection versus cancer progression, the effect of COVID‐19 vaccination in cancer patients, and the risk and prognosis of vaccine side effects in cancer patients.

In the post‐vaccination questionnaire, COVID‐19‐related history and vaccination information (inoculation date and vaccine type) were first checked, and questions subsequently focused on adverse reactions after vaccination. VRAEs were classified into three categories: VRAEs localized to the inoculation site (pain at the injection site, local erythema, and sore arm), VRAEs occurring within hours of vaccination (skin rash, angioedema, dyspnea, and anaphylactic shock), and VRAEs occurring within 7 days of vaccination (fever, chills, headache, myalgia or arthralgia, nausea or vomiting, fatigue, and abnormal bleeding). Patients were asked if they had experienced each VRAE, along with its severity (“no” or “yes, but no limitation in daily living” or “yes, interferes with daily activities” or “yes, unable to do daily activities” or” yes, required hospital treatment”) and duration (from 0 to >4 days). To assess severity more objectively, we also asked the participants whether they had been treated with each VRAE (“no” or “home‐based care with over the counter drugs” or “local primary clinic visit” or “emergency room visit” or “hemato‐oncology outpatient clinic visit earlier than original appointment” or “hospitalization”). Respondents were allowed to freely describe other unsolicited adverse events.

The electronic medical records of the study participants were reviewed to derive their clinical information, and the most recent blood test results within 30 days before and after the respective vaccination date were extracted to evaluate the change in complete blood count (CBC) profiles.

### 2.2 Ethical statement

This study was approved by the Institutional Review Board of each institution (IRB number: 2021GR0176 for Korea University Guro Hospital, 2021AN0248 for Korea University Anam Hospital, and 2021AS0159 for Korea University Ansan Hospital) and was conducted in accordance with the principles of the Declaration of Helsinki and the Guidelines for Good Clinical Practice. Written informed consent was obtained from all participants, and they agreed to provide their information, including their medical records.

### 2.3 Statistical analysis

Descriptive statistics were used to assess the patient demographics, clinical characteristics, and survey results. For statistical analysis, VRAEs were graded according to the Common Terminology Criteria for Adverse Events version 5.0. Differences in the CBC profiles before and after vaccination were evaluated using a paired *t*‐test. Univariate and multivariate logistic regression analyses using the backward elimination method were performed to investigate the risk factors associated with the development of VRAEs in patients with cancer. The results are reported as odds ratios (OR) with 95% confidence intervals (CI). All statistical analyses were performed using IBM SPSS Statistics for Windows (version 20.0; IBM Corp., Armonk, NY, USA) and graphs were generated using Microsoft Excel (Microsoft Corp., Redmond, WA, USA) or GraphPad Prism 5.0 (GraphPad Software, San Diego, CA, USA). Statistical significance was set at a *p*‐value of <0.05.

## 3 RESULTS

### 3.1 Patient perception of COVID‐19 and COVID‐19 vaccines

**FIGURE 1:**
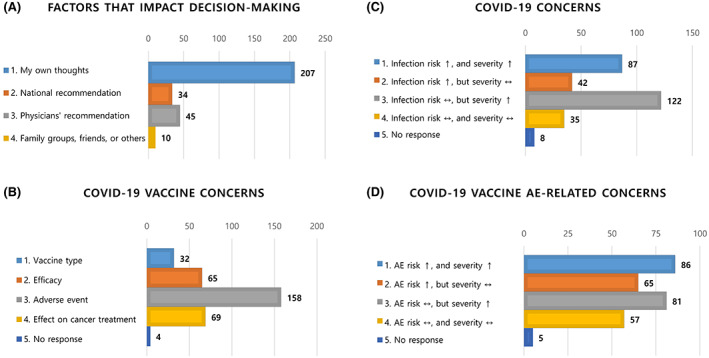
Patient perception of coronavirus disease 2019 (COVID‐19) and COVID‐19 vaccines. (A) The most important factors influencing decision‐making on vaccination. (B) The most worrisome factor when making a decision on COVID‐19 vaccination. (C) The subjective risk of COVID‐19 in cancer patients compared with that in the healthy population. (D) The subjective risk of adverse effects of COVID‐19 vaccines in cancer patients compared with that in the general population.

A total of 293 patients with hemato‐oncologic diseases from three institutions completed a perception survey on COVID‐19 and COVID‐19 vaccines. Overall, 253 (86.3%) participants were willing to be vaccinated against COVID‐19, 15 (5.1%) refused, and 25 (8.5%) had yet to decide on vaccination. Basic demographic information of the respondents is presented in Table 1. The group of patients who refused or had yet to decide on vaccination was younger and had a higher proportion of female patients than the group of patients who expressed their intention to be vaccinated. When asked about the most important factors influencing decision‐making regarding vaccination, the majority of patients selected “their own thoughts” (*n* = 207, 70.6%), followed by “physicians' recommendations” (*n* = 45, 15.4%) (Figure 1A). When deciding to undergo vaccination, patients were much more concerned about side effects than about the type or efficacy of the vaccine; 53.9% (*n* = 158) of respondents were concerned about developing VRAEs and 23.5% (*n* = 69) about negative effects on the outcomes of cancer treatment (Figure 1B). In addition, many patients believed that cancer patients were more likely to develop a serious condition after COVID‐19 infection than the general population (Figure 1C). Simultaneously, respondents were concerned that cancer patients were more likely to develop a serious condition than the general population because of vaccine‐related side effects (Figure 1D).

**TABLE 1:** Baseline characteristics of patients who responded to the survey on attitudes toward COVID‐19 vaccination

	Total (*n* = 293)	Intention to receive COVID‐19 vaccination			*p*‐value
		Yes (*n* = 253)	No (*n* = 15)	Unsure (*n* = 25)	
Sex					0.005
Male	160 (54.6%)	146 (57.7%)	8 (53.3%)	6 (24.0%)	
Female	133 (45.4%)	107 (42.3%)	7 (46.7%)	19 (76.0%)	
Age					
Years, median [IQR]	65.0 [59.0; 73.0]	66.0 [60.0; 73.0]	64.0 [52.5; 70.0]	53.0 [45.0; 64.0]	<0.001
≥60 years	214 (73.0%)	197 (77.9%)	9 (60.0%)	8 (32.0%)	<0.001
<60 years	79 (27.0%)	56 (22.1%)	6 (40.0%)	17 (68.0%)	
Diagnosis					0.538
Hematologic Malignancies	43 (14.7%)	40 (15.8%)	2 (13.3%)	1 (4.0%)	
Other Hematologic Diseases	3 (1.0%)	3 (1.2%)	0 (0.0%)	0 (0.0%)	
Solid Cancer^a^	247 (84.3%)	210 (83.0%)	13 (86.7%)	24 (96.0%)	
Stage 1	8 (3.2%)	8 (3.8%)	0 (0.0%)	0 (0.0%)	0.120
Stage 2	30 (12.1%)	23 (11.0%)	1 (7.7%)	6 (25.0%)	
Stage 3	35 (14.2%)	26 (12.4%)	4 (30.8%)	5 (20.8%)	
Stage 4	174 (70.4%)	153 (72.9%)	8 (61.5%)	13 (54.2%)	

Abbreviation: IQR, Interquartile range.

^a^ Detailed types of solid cancer are presented in Table S1.

### 3.2 VRAEs

During the study period, 255 and 186 patients participated in a safety survey after the first and second vaccine doses, respectively. Baseline characteristics, including clinicopathological data and vaccine information, are summarized in Table 2. At the time of the first and second vaccinations, 199 (78.0%) and 152 (81.7%) patients, respectively, received systemic treatment for hemato‐oncologic diseases. Treatment was classified according to the type of systemic agent administered (Table 3).

**TABLE 2:** Baseline characteristics of patients who received vaccines for COVID‐19

	First dose (*n* = 255)	Second dose (*n* = 186)
**Sex**		
Male	141 (55.3%)	109 (58.6%)
Female	114 (44.7%)	77 (41.4%)
**Age**		
Year, median [IQR]	66.0 [61.0; 73.0]	67.0 [60.0; 75.0]
≥60 years	202 (79.2%)	142 (76.3%)
<60 years	53 (20.8%)	44 (23.7%)
**Diagnosis**		
Hematologic Malignancies	40 (15.7%)	23 (12.4%)
Other Hematologic Diseases	2 (0.8%)	0 (0.0%)
Solid Cancer	213 (83.5%)	163 (87.6%)
Stage 1	13 (6.1%)	6 (3.7%)
Stage 2	27 (12.7%)	17 (10.4%)
Stage 3	26 (12.2%)	18 (11.0%)
Stage 4	147 (69.0%)	122 (74.8%)
**Comorbidities**	146 (57.3%)	113 (60.8)
Diabetes Mellitus	41 (16.1%)	33 (17.7%)
Hypertension	55 (21.6%)	45 (24.2%)
Hyperlipidemia	12 (4.7%)	8 (4.3%)
Cardiovascular Diseases	20 (7.8%)	18 (9.7%)
Respiratory Diseases	10 (3.9%)	6 (3.2%)
Other Cancers	11 (4.3%)	8 (4.3%)
Others	23 (9.0%)	15 (8.1%)
**History of COVID‐19**	5 (2.0%)	5 (2.7%)
**Vaccine Type**		
AZD1222 (Astrazeneca)	147 (58.1%)	88 (47.3%)
BNT162b2 (Pfizer/Biontech)	98 (38.7%)	92 (49.5%)
mRNA‐1273 (Morderna)	7 (2.8%)	6 (3.2%)
JNJ‐78436735 (Janssen)	1 (0.4%)	0 (0.0%)
Unknown	2 (0.8%)	0 (0.0%)

Abbreviation: IQR Interquartile range.

**TABLE 3:** Treatment information of the patients on active oncologic treatment at the time of vaccination

	First dose (*n* = 255)	Second dose (*n* = 186)
**Treatment at the time of vaccination**		
Patients on follow‐up	56 (22.0%)	34 (18.3%)
Patients on active oncologic treatment	199 (78.0%)	152 (81.7%)
**Treatment intent**		
Adjuvant/neoadjuvant	38 (19.1%)	22 (14.5%)
Definitive	4 (2.0%)	2 (1.3%)
Palliative	157 (78.9%)	128 (84.2%)
**Types of treatment**		
Chemotherapy only	77 (38.7%)	58 (38.2%)
Chemotherapy + targeted therapy	24 (12.1%)	21 (13.8%)
Chemotherapy + immunotherapy	2 (1.0%)	2 (1.3%)
Endocrine therapy only	27 (13.6%)	15 (9.9%)
Endocrine therapy + targeted therapy	8 (4.0%)	7 (4.6%)
Targeted therapy only	45 (22.6%)	34 (22.4%)
Immunotherapy only	15 (7.5%)	14 (9.2%)
Immunotherapy + targeted therapy	1 (0.5%)	1 (0.7%)

**FIGURE 2:**
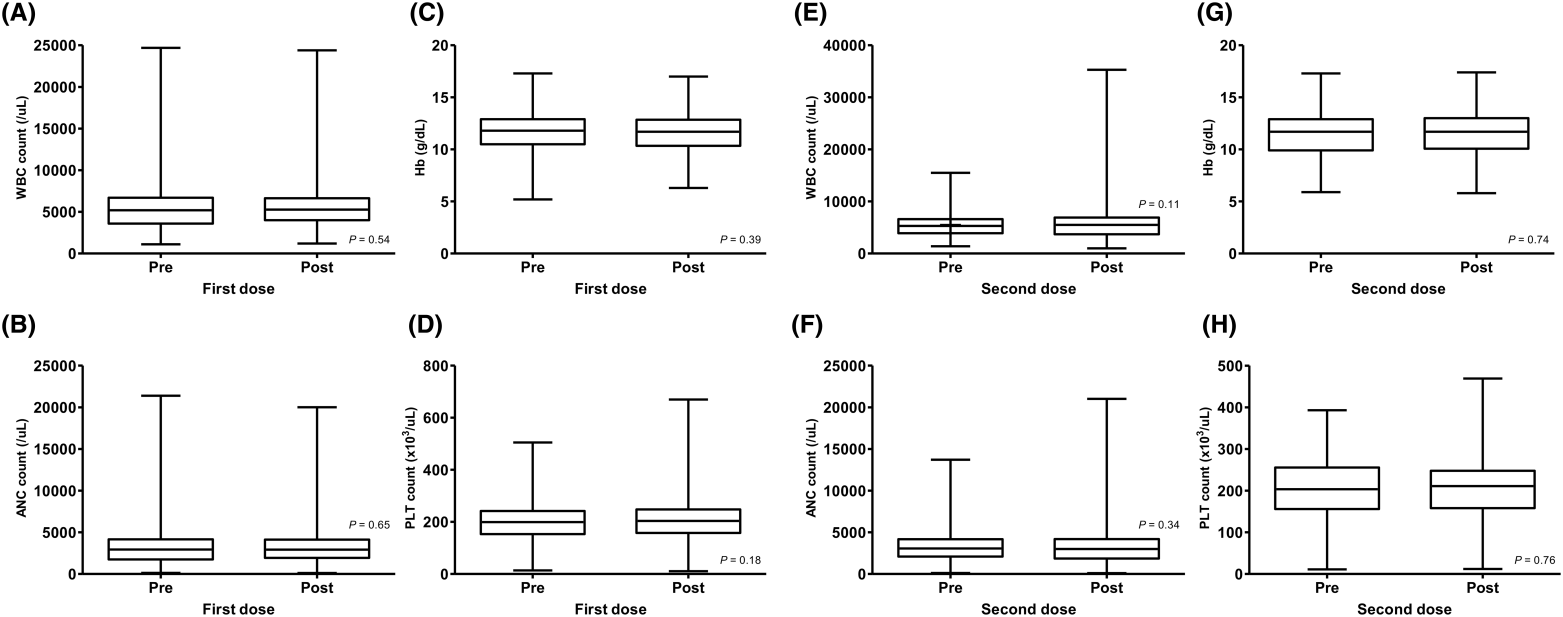
Changes in complete blood count (CBC) profiles before and after coronavirus disease 2019 (COVID‐19) vaccination. (A–D) Changes in white blood cell (WBC) count, absolute neutrophil count (ANC), hemoglobin (Hb) level, and platelet (PLT) count after the first dose compared to before. (E–F) Changes in WBC count, ANC, Hb level, and PLT count after the second dose compared to before.

Table 4 summarizes the graded VRAEs that patients reported after COVID‐19 vaccination. At the first dose, 62.0% of the patients reported any grade of VRAEs, of whom 2.4% experienced grade 3 events. Injection site pain (47.1%) and sore arm pain (26.7%) were the most common VRAEs, followed by myalgia (24.3%), fatigue (23.9%), and headache (14.5%). There were two reported cases of hospitalization, both due to anaphylaxis after vaccination. Grade 4 or 5 VRAEs were not observed. Regarding the second dose, 48.9% of the patients reported any grade of VRAEs, of whom 2.7% experienced grade 3 events. Injection site pain (36.6%) and sore arm pain (23.1%) were the most common VRAEs, followed by fatigue (18.8%), myalgia (16.7%), and headache (12.4%). Two patients with anaphylaxis requiring hospitalization after the second dose were reported, which were different from those reported after the first dose. No other patients were hospitalized for VRAEs and no grade 4 or 5 VRAEs were observed. CBC results within 30 days before and after vaccination were obtained from 157 and 118 patients based on their first and second doses, respectively. There were no significant changes in the white blood cell (WBC) count, hemoglobin (Hb) level, platelet count, or absolute neutrophil count (ANC) before and after vaccination at either dose (Figure 2).

**TABLE 4:** Adverse events reported after the first and second doses of COVID‐19 vaccines

	First dose (*n* = 255)				Second dose (*n* = 186)			
	Any grade	Grade 1	Grade 2	Grade 3	Any grade	Grade 1	Grade 2	Grade 3
**Any adverse event**	158 (62.0%)	136 (53.3%)	16 (6.3%)	6 (2.4%)	91 (48.9%)	77 (41.4%)	9 (4.8%)	5 (2.7%)
**Local adverse events**								
Pain at the injection site	120 (47.1%)	112 (43.9%)	7 (2.7%)	1 (0.4%)	68 (36.6%)	62 (33.3%)	5 (2.7%)	1 (0.5%)
Erythema	13 (5.1%)	5 (2.0%)	8 (3.1%)	0 (0.0%)	14 (7.5%)	10 (5.4%)	4 (2.2%)	0 (0.0%)
Sore arm	68 (26.7%)	62 (24.3%)	6 (2.4%)	0 (0.0%)	43 (23.1%)	41 (22.0%)	2 (1.1%)	0 (0.0%)
**Allergic/anaphylactic reactions**								
Rash	8 (3.1%)	6 (2.4%)	0 (0.0%)	2 (0.8%)	6 (3.2%)	5 (2.7%)	1 (0.5%)	0 (0.0%)
Dyspnea	2 (0.8%)	1 (0.4%)	0 (0.0%)	1 (0.4%)	2 (1.1%)	1 (0.5%)	0 (0.0%)	1 (0.5%)
Facial swelling/angioedema	4 (1.6%)	3 (1.2%)	0 (0.0%)	1 (0.4%)	2 (1.1%)	1 (0.5%)	0 (0.0%)	1 (0.5%)
Hypotension/shock	1 (0.4%)	0 (0.0%)	0 (0.0%)	1 (0.4%)	0 (0.0%)	—	—	—
**Adverse events within 7 days after vaccination**								
Fever	23 (9.0%)	20 (7.8%)	3 (1.2%)	0 (0.0%)	15 (8.1%)	13 (7.0%)	2 (1.1%)	2 (1.1%)
Chills	19 (7.5%)	16 (6.3%)	2 (0.8%)	1 (0.4%)	16 (8.6%)	12 (6.5%)	2 (1.1%)	2 (1.1%)
Headache	37 (14.5%)	31 (12.2%)	4 (1.6%)	2 (0.8%)	23 (12.4%)	19 (10.2%)	4 (2.2%)	0 (0.0%)
Myalgia/arthralgia	62 (24.3%)	56 (22.0%)	4 (1.6%)	2 (0.8%)	31 (16.7%)	29 (15.6%)	1 (0.5%)	1 (0.5%)
Nausea/vomiting	6 (2.4%)	5 (2.0%)	0 (0.0%)	1 (0.4%)	4 (2.2%)	3 (1.6%)	1 (0.5%)	0 (0.0%)
Fatigue	61 (23.9%)	55 (21.6%)	5 (2.0%)	1 (0.4%)	35 (18.8%)	29 (15.6%)	3 (1.6%)	3 (1.6%)
Abnormal bleeding	0 (0.0%)	—	—	—	0 (0.0%)	—	—	—

Excluding patients with benign hematologic disease (*n* = 2) and incomplete datasets (*n* = 2, missing data for vaccine type), individuals were further analyzed for their risk of developing any grade of VRAEs. Multivariable analysis showed that female sex (OR, 2.35; 95% CI, 1.32–4.28; *p* = 0.004), age < 60 years (OR, 3.76; 95% CI, 1.59–10.02; *p* = 0.004), and active cancer treatment (OR, 2.29; 95% CI, 1.13–4.80; *p* = 0.024) were independent risk factors for VRAE development after the first dose. Similarly, female sex (OR, 4.02; 95% CI, 2.03–8.19; *p* < 0.001), active cancer treatment (OR, 3.39; 95% CI, 1.41–8.82; *p* = 0.008), and messenger ribonucleic acid (mRNA) vaccines (OR, 2.97; 95% CI, 1.54–5.90; *p* = 0.001) were shown to be independent risk factors for VRAE development at the second dose (Table 5). Notably, the administration of immuno‐oncology (IO) agents was not associated with a risk of developing VRAE (Table S2).

**TABLE 5:** Univariate and multivariable (stepwise backward) logistic regression analyses for risk factors for vaccine‐related adverse events

Covariates	First dose						Second dose					
	Univariate			Multivariable			Univariate			Multivariable		
	OR	95% CI	*p*‐value	OR	95% CI	*p*‐value	OR	95% CI	*p*‐value	OR	95% CI	*p*‐value
**Sex**												
Male	Ref			Ref			Ref			Ref		
Female	2.95	1.73–5.13	<0.001	2.35	1.32–4.28	0.004	4.13	2.24–7.79	<0.001	4.02	2.03–8.19	<0.001
**Age**												
≥60 years	Ref			Ref			Ref			—	—	—
<60 years	5.20	2.37–13.13	<0.001	3.76	1.59–10.02	0.004	2.49	1.25–5.16	0.011	—	—	—
**Comorbidities**												
None	Ref			—	—	—	Ref			Ref		
≥1	0.48	0.28–0.82	0.007	—	—	—	0.43	0.23–0.78	0.006	0.57	0.28–1.16	0.118
**Diagnosis**												
Hematologic Malignancy	Ref			—	—	—	Ref			—	—	—
Solid Cancer	1.40	0.70–2.76	0.339	—	—	—	1.57	0.65–3.97	0.318	—	—	—
**Stage**	0.70	0.50–0.95	0.027	0.63	0.42–0.92	0.019	1.08	0.75–1.57	0.680	—	—	—
**On treatment**												
None	Ref			Ref			Ref			Ref		
Yes	1.86	1.01–3.42	0.047	2.29	1.13–4.80	0.024	3.25	1.47–7.80	0.005	3.39	1.41–8.82	0.008
**Vaccine type**												
Vector	Ref			Ref			Ref			Ref		
mRNA	2.17	1.27–3.74	0.005	1.76	0.96–3.26	0.069	2.42	1.35–4.39	0.003	2.97	1.54–5.90	0.001

Abbreviations: OR, Odds ratio; CI, Confidence interval; mRNA, messenger ribonucleic acid; Ref, Reference.

## 4 DISCUSSION

In this study, we examined the perception of the COVID‐19 vaccine among patients with cancer in Korea and the actual side effects that occurred after vaccination. The Korean patients with cancer in this study had a relatively positive attitude toward COVID‐19 vaccination. The proportion of participants in our survey who showed vaccine hesitancy, who either refused to undergo vaccination or had not yet decided was 13.6%, similar to or lower than the proportions reported in other countries,^16^, ^17^, ^18^ even compared with those in previous studies conducted in early 2021 in Korea.^9^ For reference, the domestic vaccination rate at the end of the study period, in November 2021, was 81.7% for the first dose and 78.1% for the second dose.^19^ Previous studies have indicated that patients being treated for cancer^16^ and those who considered themselves more vulnerable to COVID‐19^17^ have a higher acceptance rate for COVID‐19 vaccination. In addition, those with vaccine hesitancy were often concerned that the COVID‐19 vaccine could negatively affect the prognosis of cancer.^18^ Considering that the majority of patients in this study had a relatively advanced stage of the disease and approximately 80% of patients were receiving active cancer treatment, it could be interpreted that the Korean patients in this study were more concerned about their cancer treatment being affected by COVID‐19 than about the COVID‐19 vaccines themselves.

More than half of the patients responded that they were concerned about the development of VRAEs. The majority of patients also considered themselves more likely to develop a serious condition than the general population with VRAE. However, the most commonly reported local and systemic VRAEs in this study were mild‐to‐moderate in severity, and the actual incidence of VRAEs of all grades was 62% and 49% after the first and second doses, respectively. These values are lower than those reported in a global phase 3 study conducted on a healthy population, wherein more than 70% of the participants reported experiencing local or systemic VRAEs after AZD1222 or BNT162b2 vaccination.^20^, ^21^ This is in line with the result of a previous study demonstrating that notably fewer patients with cancer reported moderate symptoms compared with healthy controls after receiving the first and second doses of BNT162b2.^22^ In addition, in a review of several studies, Tran et al. showed that the safety profiles of different types of COVID‐19 vaccines were comparable or even better among patients with cancer than in healthy controls.^10^ An overlap in symptoms with chronic diseases, anti‐cancer therapies, and reduced immunogenicity have been suggested as possible reasons for the development of fewer VRAEs in cancer patients.^23^

We observed that the risk of VRAE development was higher in those who were female, younger, currently being treated, or vaccinated with mRNA vaccines. It has been reported that the risk of the development of adverse events following vaccination is generally higher in females,^24^ and this has consistently been noted following COVID‐19 vaccination. In the United States, 79.1% of adverse events reported in the first month of COVID‐19 vaccine safety monitoring were in females.^25^ Prior studies in Israel have indicated that the incidence of adverse events following the administration of the Pfizer‐BioNTech COVID‐19 vaccine was substantially higher in females than in males.^26^ Thromboembolic events reported in Europe following the administration of the AstraZeneca‐COVID‐19 vaccine were predominantly in females.^27^ Although the exact mechanisms underlying the higher risk of VRAEs in females are not fully understood, sex differences in immunity influenced by X chromosome‐linked genes, sex hormones, and microbiomes have been suggested as possible biological factors mediating these differences in response to vaccines.^26^, ^28^ The trend of a high incidence of VRAEs in younger people has already been reported from clinical trial data of different types of COVID‐19 vaccines.^29^, ^30^, ^31^ A robust immune response in young people is suggested as a reason for the adverse effects of the vaccines, which are unlikely to be different for cancer patients.

Consistent with a prior study,^22^ no differences were observed in the risk of VRAE development in patients with hematological or solid malignancies. Notably, patients who were actively receiving cancer treatment reported more frequent VRAEs than those not undergoing treatment at the time of vaccination. However, most had mild‐to‐moderate symptoms that did not affect the progress of scheduled anti‐cancer treatments. The systemic treatment regimens that our patients received were very diverse, including a multitude of single‐agent and combination therapies, but no obvious difference in VRAEs according to the type of systemic treatment was noted. According to a prior study conducted in the UK, receiving chemotherapy during the vaccination period did not influence the risk of VRAE development.^23^ Regarding the possible interactions between immunotherapy and COVID‐19 vaccines,^32^, ^33^ as shown in a previous report of the BNT162b2 COVID‐19 vaccine,^34^ receiving IO agents was not a risk factor for VRAE development in this study.

Our results indicate that vaccination with non‐mRNA vaccines should be considered for cancer patients to minimize the occurrence of VRAE. Previous studies have shown that the BBIBP‐CorV, an inactivated vaccine that was not introduced in Korea, was also a tolerable and effective method for preventing COVID‐19 in patients with cancer.^11^, ^12^ However, as this was not a head‐to‐head comparison and the overall frequency or severity of VRAEs was very low, further studies are needed to identify the most suitable COVID‐19 vaccine for patients with cancer.

The patients enrolled in this study had records of blood tests conducted at the respective clinics periodically for cancer treatment or follow‐up, and we were able to investigate hematologic toxicities, such as vaccine‐induced immune thrombotic thrombocytopenia (VITT). VITT is one of the VRAEs of special interest that has been monitored and reported periodically by the Korea Centers for Disease Control and Prevention. From February 2021 to September 2022, 213 suspected cases of VITT were reported in Korea, of which four cases were confirmed to have a causal relationship. Among these, two cases were of cerebral vein thrombosis (CVT), one was of deep vein thrombosis, and one was of pulmonary embolism. Both cases of CVT occurred in men in their 30s.^35^ Although it should be considered that blood was not drawn at a fixed time after vaccination and many patients were undergoing chemotherapy, no case of VITT was observed, and there were no significant changes in overall CBC profiles before and after vaccination in this study.

A major limitation of this study is its retrospective design. Nevertheless, given that a considerable number of patients visited clinics frequently for regular treatment, most of the patients participated in the survey relatively closely after vaccination, therefore, the memory bias was probably low. In addition, our patient cohort was heterogeneous with regard to the primary cancer diagnosis and systemic treatment that the patients received, which may have obscured subtle differences in VRAEs related to different types of cancer and chemotherapeutic agents. Finally, we did not address the efficacy of COVID‐19 vaccines and their immunogenicity in cancer patients, such as serological responses. It was difficult to prospectively collect blood samples from the patients because they were vaccinated on a date they desired at a nationally designated vaccine center instead of at our institute. Instead, we planned to track changes in the CBC profiles of each patient as they underwent blood tests regularly in the outpatient clinic. Previous studies have demonstrated blunted seropositivity in patients with hematological and/or solid malignancies^4^, ^13^, ^36^ and in those undergoing cytotoxic chemotherapy.^37^, ^38^ Moreover, owing to the short observation time, there is currently very limited knowledge on the long‐term adverse effects of COVID‐19 vaccines in individuals with and without cancer.^39^ Therefore, although rare, the possibility of long‐term health complications from vaccination should be considered.

## 5 CONCLUSIONS

Overall, our study indicated that COVID‐19 vaccines were well tolerated and no severe adverse effects were observed in cancer patients, in agreement with the reports of previous studies. Most studies so far have been conducted on Western patients, and we demonstrated that there were no significant ethnic differences in the toxicity profiles of COVID‐19 vaccines in Korean cancer patients compared with those in Western patients. However, as shown in the results of the perception survey, there was a high prevalence of fear related to vaccine‐associated adverse effects. Based on our results, we should reassure patients about the safety of COVID‐19 vaccines. In addition, prospective clinical studies for vulnerable populations, such as patients with cancer, should be conducted when developing novel vaccines to establish firm guidelines for appropriate management.

## AUTHOR CONTRIBUTIONS

**Kyoungmin Lee:** Conceptualization (equal); data curation (equal); formal analysis (equal); methodology (equal); writing – original draft (equal). **In Hae Park:** Data curation (equal); resources (equal); supervision (equal). **Sang Cheul Oh:** Data curation (equal); resources (equal); supervision (equal). **Jae Hong Seo:** Data curation (equal); resources (equal); supervision (equal). **Min Ji Jeon:** Data curation (equal); resources (equal); supervision (equal). **Eun Sang Yu:** Data curation (equal); resources (equal); supervision (equal). **Daesik Kim:** Data curation (equal); resources (equal); supervision (equal). **Chul Won Choi:** Data curation (equal); resources (equal); supervision (equal). **Ah‐reum Lim:** Data curation (equal); formal analysis (equal); investigation (equal); resources (equal). **Myung Han Hyun:** Data curation (equal); formal analysis (equal); investigation (equal); resources (equal). **Ju Won Kim:** Data curation (equal); formal analysis (equal); investigation (equal); resources (equal). **Jwa Hoon Kim:** Data curation (equal); formal analysis (equal); investigation (equal); resources (equal). **Yoon Ji Choi:** Conceptualization (equal); investigation (equal); project administration (equal); supervision (equal); validation (equal). **Soohyeon Lee:** Data curation (equal); resources (equal); supervision (equal). **Kyong Hwa Park:** Data curation (equal); resources (equal); supervision (equal). **Yeul‐Hong Kim:** Data curation (equal); resources (equal); supervision (equal). **Jung Yoon Choi:** Data curation (equal); formal analysis (equal); investigation (equal); resources (equal). **Jung Sun Kim:** Conceptualization (equal); investigation (equal); project administration (equal); supervision (equal); validation (equal). **Se Ryeon Lee:** Data curation (equal); resources (equal); supervision (equal). **Hwa Jung Sung:** Data curation (equal); resources (equal); supervision (equal). **Eun Joo Kang:** Conceptualization (equal); data curation (equal); methodology (equal); project administration (equal); writing – review and editing (equal).

## CONFLICTS OF INTEREST

There are no conflicts of interest relevant to this article to report.

## ETHICS APPROVAL STATEMENT

This study was approved by the institutional review board of each institution (IRB number:2021GR0176 for Korea University Guro Hospital, 2021AN0248 for Korea University Anam Hospital, and 2021AS0159 for Korea University Ansan Hospital). Written informed consent was obtained from all participants, and all agreed to provide their information, including their medical records.

## Supporting information


Table S1
Table S2

## Data Availability

The original contributions presented in the study are included in the article/supplementary material, further inquiries can be directed to the corresponding authors.
