# Supplementary material for: Perception and safety analysis of COVID‐19 vaccination in cancer patients: A multicenter, real‐world study
Source: Cancer Med. 2022 Nov 13;12(5):5558–68. doi: 10.1002/cam4.5400 (PMC9877582; doi:10.1002/cam4.5400)
Supplement: Supplementary file 1 — Table S1 Table S2 [file CAM4-12-5558-s001.docx]

**Supplementary Table S1. Types of solid cancer in this study**

| **Type of cancer** | **Total**  **(n = 247)** |
| --- | --- |
|  |  |
| Breast cancer | 64 (25.9%) |
| Colorectal cancer | 36 (14.6%) |
| Lung cancer | 34 (13.8%) |
| Head and neck cancer | 16 (6.5%) |
| Gastrointestinal stromal tumor | 14 (5.7%) |
| Prostate cancer | 13 (5.3%) |
| Stomach cancer | 13 (5.3%) |
| Biliary tract cancer | 8 (3.2%) |
| Sarcoma | 8 (3.2%) |
| Urothelial cancer | 8 (3.2%) |
| Esophageal cancer | 7 (2.8%) |
| Melanoma | 5 (2.0%) |
| Neuroendocrine tumor | 4 (1.6%) |
| Ovarian cancer | 4 (1.6%) |
| Skin cancer | 3 (1.2%) |
| Thymoma | 3 (1.2%) |
| Pancreas cancer | 2 (0.8%) |
| Anal cancer | 1 (0.4%) |
| Liver cancer | 1 (0.4%) |
| Medullary thyroid cancer | 1 (0.4%) |
| Kidney cancer | 1 (0.4%) |
| Vulvar cancer | 1 (0.4%) |

**Supplementary Table S2. Univariate and multivariate (stepwise backward) logistic regression analyses of risk factors for vaccine-related adverse events in patients undergoing active cancer treatment**

| **Covariates** | **First dose** | | | | | | **Second dose** | | | | | |
| --- | --- | --- | --- | --- | --- | --- | --- | --- | --- | --- | --- | --- |
|  | **Univariate** | | | **Multivariable** | | | **Univariate** | | | **Multivariable** | | |
|  | **OR** | **95% CI** | **p-value** | **OR** | **95% CI** | **p-value** | **OR** | **95% CI** | **p-value** | **OR** | **95% CI** | **p-value** |
| **Sex** |  |  |  |  |  |  |  |  |  |  |  |  |
| Male | Ref |  |  | - | - | - | Ref |  |  | Ref |  |  |
| Female | 2.85 | 1.55–5.37 | <0.001 | - | - | - | 3.69 | 1.87–7.50 | <0.001 | 2.83 | 1.32–6.23 | 0.008 |
| **Age** |  |  |  |  |  |  |  |  |  |  |  |  |
| ≥ 60 years | Ref |  |  | Ref |  |  | Ref |  |  | - | - | - |
| < 60 years | 4.43 | 1.89–12.23 | 0.001 | 2.59 | 0.98–7.76 | 0.067 | 3.11 | 1.42–7.28 | 0.006 | - | - | - |
| **Comorbidities** |  |  |  |  |  |  |  |  |  |  |  |  |
| None | ref |  |  | ref |  |  | ref |  |  | ref |  |  |
| ≥1 | 0.40 | 0.21–0.73 | 0.003 | 0.40 | 0.19–0.81 | 0.012 | 0.39 | 0.20–0.75 | 0.006 | 0.42 | 0.19–0.92 | 0.033 |
| **Diagnosis** |  |  |  |  |  |  |  |  |  |  |  |  |
| Hematologic Malignancy | Ref |  |  | - | - | - | Ref |  |  | - | - | - |
| Solid Cancer | 1.59 | 0.69–3.61 | 0.272 | - | - | - | 1.36 | 0.49–3.84 | 0.547 | - | - | - |
| **Stage** | 0.39 | 0.19–0.66 | 0.003 | 0.36 | 0.17–0.64 | 0.002 | 0.79 | 0.46–1.27 | 0.344 | - | - | - |
| **Immunotherapy** |  |  |  |  |  |  |  |  |  |  |  |  |
| No | Ref |  |  | - | - | - | Ref |  |  | Ref |  |  |
| Yes | 0.65 | 0.24–1.77 | 0.382 | - | - | - | 0.31 | 0.10–0.90 | 0.039 | 0.31 | 0.09–0.97 | 0.053 |
| **Vaccine type** |  |  |  |  |  |  |  |  |  |  |  |  |
| Vector | Ref |  |  | Ref |  |  | Ref |  |  | Ref |  |  |
| mRNA | 1.94 | 1.06–3.60 | 0.033 | 2.19 | 1.06–4.62 | 0.036 | 2.45 | 1.28–4.76 | 0.007 | 3.18 | 1.55–6.79 | 0.002 |

*OR* Odds ratio, *CI* Confidence interval, *mRNA* messenger ribonucleic acid; *Ref*, Reference
